# Examining the replicability of backfire effects after standalone corrections

**DOI:** 10.1186/s41235-023-00492-z

**Published:** 2023-07-03

**Authors:** Toby Prike, Phoebe Blackley, Briony Swire-Thompson, Ullrich K. H. Ecker

**Affiliations:** 1 School of Psychological Science, University of Western Australia, Perth, Australia; 2 Network Science Institute, Northeastern University, Boston, USA; 3 Institute of Quantitative Social Science, Harvard University, Cambridge, USA; 4 Public Policy Institute, University of Western Australia, Perth, Australia

**Keywords:** Misinformation, Corrections, Familiarity backfire effect, Skepticism

## Abstract

Corrections are a frequently used and effective tool for countering misinformation. However, concerns have been raised that corrections may introduce false claims to new audiences when the misinformation is novel. This is because boosting the familiarity of a claim can increase belief in that claim, and thus exposing new audiences to novel misinformation—even as part of a correction—may inadvertently increase misinformation belief. Such an outcome could be conceptualized as a *familiarity backfire effect*, whereby a familiarity boost increases false-claim endorsement above a control-condition or pre-correction baseline*.* Here, we examined whether standalone corrections—that is, corrections presented without initial misinformation exposure—can backfire and increase participants’ reliance on the misinformation in their subsequent inferential reasoning, relative to a no-misinformation, no-correction control condition. Across three experiments (total *N* = 1156) we found that standalone corrections did not backfire immediately (Experiment 1) or after a one-week delay (Experiment 2). However, there was some mixed evidence suggesting corrections may backfire when there is skepticism regarding the correction (Experiment 3). Specifically, in Experiment 3, we found the standalone correction to backfire in open-ended responses, but only when there was skepticism towards the correction. However, this did not replicate with the rating scales measure. Future research should further examine whether skepticism towards the correction is the first replicable mechanism for backfire effects to occur.

**Supplementary Information:**

The online version contains supplementary material available at 10.1186/s41235-023-00492-z.

## Significance statement

Belief in false claims and relying on misinformation in one’s reasoning and decision making can have wide-ranging negative consequences for both individuals and society. Therefore, it is crucial to find effective tools to counter misinformation and to ensure those tools do not inadvertently increase belief in the misinformation. Corrections are one of the most common tools used for tackling misinformation, with individuals and organizations regularly issuing fact-checks or correcting news stories as information becomes available. There is considerable research showing that corrections effectively reduce misinformation belief and reliance. However, concerns have been raised that if—as part of a correction—misinformation is spread to new audiences, then this may lead to greater misinformation reliance. We conducted a series of three experiments (total *N* = 1156) to test whether standalone corrections—that is, corrections presented without initial misinformation exposure—are at risk of backfiring and increasing misinformation reliance. Corrections did not backfire when misinformation reliance was measured immediately (Experiment 1) or after a one-week delay (Experiment 2). However, when we intentionally chose scenarios to induce skepticism in the correction (Experiment 3), there was some mixed evidence that corrections may backfire. Future research should further examine whether correction skepticism reliably leads to backfire effects. For now, we advise those combating misinformation to continue to use corrections as part of their toolkit. As long as people are not skeptical of the correction, there is low risk that corrections targeting novel misinformation or reaching new audiences will backfire.

## Introduction

Misinformation—false or misleading information potentially believed to be true—presents a significant societal challenge (Ecker et al., 2022). Misinformation about health (e.g., “doctor dies following COVID vaccination”; Widmer, 2021) or politics (e.g., “the 2020 US election was stolen”; Cassidy, 2021) can negatively impact both individuals and society (Ha et al., 2021; Horne et al., 2015; Lewandowsky et al., 2017; MacFarlane et al., 2021; Swire-Thompson & Lazer, 2022; Thorson, 2016). It is therefore crucial to develop effective interventions for countering misinformation. Corrections are one of the most widely used and studied interventions, with research clearly indicating that they are an effective intervention for reducing misconceptions and misinformed reasoning and decision making (Lewandowsky et al., 2020; Paynter et al., 2019). That being said, it is also clear that corrections are generally only partially effective, with considerable evidence showing that people continue to rely on misinformation in their reasoning even after being given corrections. This continued reliance on misinformation has been termed the *continued influence effect* (Johnson & Seifert, 1994; for a review see Ecker et al., 2022).

Beyond corrections not being fully efficacious, an even greater concern has been that under certain conditions, corrections can be entirely ineffective or may even backfire, resulting in increased misinformation reliance (Lewandowsky et al., 2012). The current evidence suggests that this is a rare phenomenon that can occur if a correction attacks a worldview-bolstering belief (i.e., the *worldview backfire effect*; Nyhan & Reifler, 2010; but see Ecker & Ang, 2019; Ecker et al., 2021; Wood & Porter, 2019). A second type of backfire effect that has been proposed is a familiarity-driven effect (Lewandowsky et al., 2012; Schwarz et al., 2007). Specifically, if a correction repeats the misinformation in order to invalidate it, the repetition of the false claim may boost its familiarity and thus inadvertently increase claim belief (Pluviano et al., 2017, 2019; Skurnik et al. 2007 [unpublished; discussed in Schwarz et al., 2007]). However, there is little empirical evidence in support of this *familiarity backfire effect* (Cameron et al., 2013; Ecker et al., 2017; Ecker et al., 2020c; Ecker et al., 2023; Kemp et al., 2022a, 2022b; Swire et al., 2017; Wahlheim et al., 2020; for reviews, see Ecker et al., 2022; Swire-Thompson et al., 2022).

Despite this relative lack of evidence, it has been proposed that there are several situations where familiarity backfire effects may be especially likely to occur. One situation is when a person encounters a correction that negates a novel piece of misinformation (Schwarz et al., 2016). Put simply, a person learning that “*x* did not happen” may develop a stronger belief in “*x*” than someone who was never given the *x*-denying correction (or any other information about *x*). This may be due to the correction boosting the familiarity of the novel claim. Indeed, a recent study by Autry and Duarte (2021) found that presenting participants with a correction backfired when they had not been exposed to the initial, novel misinformation. In other words, a standalone correction seemed to cause greater misinformation reliance relative to a situation where participants were not exposed to the misinformation nor the correction. A second occasion where such an effect may be likely to arise is if people are particularly skeptical of the correction. For instance, the fact that a correction is issued may be interpreted as evidence that the misinformation was once believed to be true or is believed to be true by some people. Therefore, in the current study we sought to conceptually replicate the findings of Autry and Duarte (2021), to ascertain whether corrections can continue to be safely used even if people may have not encountered the targeted piece of misinformation before.

Theoretical accounts of the continued influence effect may also provide insight into why corrections may potentially backfire. The two dominant accounts of continued influence are the mental-model account and the selective-retrieval account. The mental-model account posits that people desire a complete mental model of an event and its associated cause (Johnson & Seifert, 1994). Therefore, people may be motivated to continue to rely on false information post-correction because it allows them to retain a complete mental model of the event and avoid the psychological discomfort associated with an incomplete mental model (Ecker et al., 2011; Susmann & Wegener, 2022). When encountering a standalone correction (e.g., drug use did not cause an athlete’s suspension), readers learn that an event (the athlete’s suspension) has occurred, without receiving a validated cause. As such, some people may increase their belief in the negated information (drug use) to form and retain a complete mental model that includes a cause of the event, even though they were exposed to the cause only as part of a correction negating it.

The second account of the continued influence effect proposes that misinformation and corrective information are concurrently stored in memory (Ayers & Reder, 1998), and that continued influence is caused by the selective retrieval of misinformation (Ecker et al., 2010). One variant of this account is based on dual-process theories of memory, which assume a rapid, automatic retrieval process driven by familiarity, and a slow, strategic retrieval process required to recollect contextual details, including information source and veracity (Yonelinas, 2002). According to this account, continued influence can arise if misinformation is automatically retrieved based on its familiarity, and strategic recollection of corrective information fails. It follows that misinformation familiarity can be a driver of continued influence, which is in line with the *illusory truth effect*, the finding that the more familiar a piece of information is, the more likely it is perceived as true (Begg et al., 1992; De keersmaecker et al., 2020; Dechêne et al., 2010; Fazio et al., 2015; Pennycook et al., 2018; Unkelbach, 2007). Because corrections typically repeat the misinformation (e.g., the correction “the athlete’s suspension was not caused by a failed drug test” inevitably repeats the two concepts “suspension” and “drug” and their association), presenting a correction without initial misinformation exposure may boost the familiarity of the misinformation compared to baseline, increasing the subsequent likelihood of misinformation being retrieved and relied upon.

Although these accounts offer some theoretical justification for why standalone corrections may backfire, some previous studies using standalone corrections without initial misinformation exposure have not found any evidence of deleterious effects (Ecker et al., 2020b, 2020c; Gordon et al., 2019). However, Autry and Duarte (2021) argued that the reason for this is that those studies used corrections that were *licensed* negations. A licensed negation is one that counters either a known (e.g., based on common knowledge or previous exposure) or an easily activated claim (e.g., a stereotype; Mayo et al., 2004). Therefore, because previous studies either corrected a common stereotype (e.g., that a robber was not Black; Gordon et al., 2019) or used a fact-checking approach that presented the false statement in an affirmative format together with a false tag (e.g., “Hospitals are busier on full moons—FALSE”; Ecker et al., 2020b, 2020c), Autry and Duarte suggested that the corrections were licensed.

When a licensed negation is presented, it is relatively easy for people to understand why the negation is being presented and what it is referring to (i.e., what claim is being corrected and why). This is because the specific claim being negated is either known or stereotypical. However, Autry and Duarte (2021) argued that *unlicensed* corrections—those that negate a piece of information that is unexpected or novel (Mayo et al., 2004) may be at greater risk of backfiring. This is because unlicensed corrections may require more processing, as they negate an unexpected or novel piece of information. This greater level of processing may mean that unlicensed negations are at greater risk of boosting the familiarity of the corrected misinformation than licensed corrections (Autry & Levine, 2012), thereby increasing the likelihood that the misinformation will later be selectively retrieved and relied upon.

Accordingly, Autry and Duarte (2021) ensured their misinformation was not stereotypical, and their negating corrections were not presented as tagged affirmative claims. They presented participants with a multi-paragraph passage in which participants either were or were not exposed to initial misinformation (e.g., “he saw a blue car”). Participants then received a correction (“the car was not blue”), replacement (“the car was red”), or no correction (“the car was his neighbor’s new vehicle”). Autry and Duarte found that unlicensed standalone corrections significantly increased misinformation reliance relative to a no-misinformation, no-correction condition. However, it should be noted that this finding was based on a single event report and that the effect was no longer statistically significant in a second experiment that used a broader range of materials.

Nevertheless, this finding raises the possibility that, unlike licensed negations, unlicensed negations of novel misinformation might be at unique risk of backfiring. However, before accepting this conclusion, it is important to establish that unlicensed negations reliably lead to backfire effects. Moreover, to be relevant to the real world, it is also important to establish that such effects occur when unlicensed negations correct information that carries some relevance. Some corrections used by Autry and Duarte (2021) negated arbitrary side details (e.g., that a dining table was “not square”) which may be less well-remembered and less relevant to meaningful, real-world corrections than an unlicensed negation of a more central and important piece of information (e.g., the cause of an event). Additionally, presenting unlicensed negations of arbitrary side details may be perceived as odd because it violates Gricean maxims of communication (Grice, 1975). Specifically, information relevance is essential to effective communication, and therefore referring to a “big table which turned out to be not square” when a table had never previously been mentioned may be perceived as unexpected or odd by readers. Such norm violations may lead readers to appraise the information in unintended ways. For example, if the information seems entirely irrelevant, it seems particularly plausible to assume that it is only being mentioned (in a negation format) because there is some reason to believe it is true. Therefore, rather than reflecting a familiarity backfire effect, Autry and Duarte’s findings may instead be the result of communication-norm violations leading participants to infer that there are unmentioned reasons to believe the misinformation or to be skeptical of the correction.

### The present study

The current study examined the potential for unlicensed negations to backfire while ensuring that the negations referred to core (causal) event details and were not perceived as odd by participants. As in Autry and Duarte (2021), we used multi-paragraph passages, although our reports were somewhat shorter in length (approx. 200–250 words vs. 420–500). A set of eight news reports were developed and pilot-tested to ensure that the reports selected for inclusion featured causal misinformation that was not highly stereotypical (nor highly unexpected), and was not perceived as odd in the context of a standalone correction. Additionally, because we were interested in the effect of presenting (vs. not presenting) standalone corrections, we did not include the replacement condition that replaced the target misinformation with an alternative (e.g., “blue” being replaced with “red”; for further details see Autry & Duarte, 2021). Experiment 1 was a conceptual replication of Autry and Duarte (2021), which used these newly developed and tested materials to examine whether unlicensed negations of novel misinformation would backfire and increase misinformation reliance. Experiments 2 and 3 then further examined two key factors that may increase the risk of corrections backfiring, namely a delay between exposure and test, and skepticism regarding the correction, respectively.

In Experiments 1 and 2, participants were (or were not) exposed to initial misinformation, and then were (or were not) presented with a misinformation-negating correction, creating four within-subject conditions: misinformation/no-correction, misinformation/correction, no-misinformation/correction, and no-misinformation/no-correction (control). In Experiment 3, participants were never initially exposed to misinformation, with standalone corrections directly contrasted with the control condition. The extent to which participants relied on the misinformation in their event-related inferential reasoning was measured via questionnaire.

We expected participants in the misinformation/no-correction condition to have the highest level of misinformation reliance.^1^ In line with previous research (Ecker et al., 2017; Ecker et al., 2020b, 2020c; Gordon et al., 2019) we expected a correction that negates a previously presented piece of misinformation (misinformation/correction condition) to reduce but not entirely eliminate misinformation reliance (i.e., we expected a continued influence effect to emerge). Given that there is a large body of evidence demonstrating that corrections do not backfire (Cameron et al., 2013; Ecker et al., 2017, 2023; Ecker et al., 2020c; Kemp et al., 2022a, 2022b; Swire et al., 2017; Wahlheim et al., 2020) and the inconsistent results in Autry and Duarte (2021), we did not expect standalone corrections to backfire, and thus predicted misinformation reliance in the no-misinformation/correction condition to not be significantly higher than control.

## Experiment 1

### Method

Experiment 1 used a 2 × 2 within-subjects design with the independent variables of misinformation exposure (no misinformation; misinformation) and correction (no correction; correction). The dependent variable, reliance on misinformation, was measured by open-ended responses to event-summary and inference questions. Memory for report details was measured with multiple-choice questions. The experiment used a Qualtrics survey (Qualtrics, Provo, UT, USA, 2022) and was administered online.

#### Participants

Based on an a-priori power analysis (G*Power 3.1; Faul et al., 2007), a minimum sample size of 200 participants was required to detect an interaction effect of size ƒ = 0.20 (with α = 0.05 and 1 – β = 0.80).^2^ This effect size was chosen because it is the effect size used in the power analysis reported by Autry and Duarte (2021). This effect size is also consistent with recommendations by Brysbaert (2019), which suggest that Cohen’s *d* of 0.4 (*f* = 0.2) is a good first estimate of the smallest effect size of interest in psychological research. To account for potential exclusions and ensure ample statistical power, 283 participants were recruited from the online testing platform Amazon Mechanical Turk (MTurk) via CloudResearch (Litman et al., 2017). Participants were eligible if they resided in the United States of America and had previously completed more than 5000 MTurk tasks (HITs) with a minimum approval rating of 97%. The data were screened using a-priori criteria to exclude any participants who did not report their English proficiency as at least “good” (> 2 on a 5-point scale ranging from 1, *poor* to 5, *excellent*; *n* = 0), indicated they did not reside in the U.S. (*n* = 0), self-nominated their data to be excluded because of low effort (*n* = 2), provided uniform responses (*n* = 1), or did not meet the minimum memory score to ensure adequate encoding of materials (*n* = 4; see details below). The final sample size for analysis was thus *N* = 276. The sample included 133 females, 141 males, 1 non-binary individual, and 1 individual preferring not to declare their gender. Participant age ranged from 18 to 77 years (*M* = 43.04; *SD* = 11.65).^3^ The experiment took approximately 15 min to complete; participants were paid US$2.50 for their participation.

#### Materials

##### News reports

Eight novel news reports were created and pilot-tested for the current study, leading to the selection of four news reports for inclusion in Experiment 1 (see Additional file 1). In the pilot test, independent samples of *N* = 100 MTurk participants rated the reports on cause stereotypicality and oddness of a standalone correction, respectively, on a 0 to 10 rating scale (see Additional file 1 for full details). The four reports with lowest cause stereotypicality and standalone-correction oddness were selected for inclusion in Experiment 1. Each report described a fictional event; for example, one report detailed the exclusion of a football club’s star player from an important match (*“*FC Tokyo’s left winger Yasuto Tanaka has been side-lined for next Wednesday’s J1-League game”); the others related to a local government budget deficit, flight delays, and a server crash. Each report existed in four versions, depending on whether or not it contained misinformation and whether or not it contained a correction (see Table 1 for an example). In the report versions containing misinformation, the first section of the report provided a cause of the event (e.g., “It is believed that Tanaka’s exclusion is due to a failed drug test”); in the no-misinformation versions, the cause was replaced with a neutral, arbitrary statement (e.g., “It is believed that there will be a record crowd for the much-anticipated game”). Irrespective of whether the report provided misinformation initially, the report versions containing a correction provided a negating correction in the second section (e.g., “At today’s press conference the team chairman explained that Tanaka’s exclusion was not due to a failed drug test”); in the no-correction versions, this was replaced with a neutral statement (e.g., “At today’s press conference the team chairman explained that the team still had high hopes of winning the title”). The no-misinformation/no-correction control condition thus simply reported the event without mentioning any cause. Each news report was presented in two parts, on successive screens.

**Table 1:** Example scenario: athlete sidelined

All conditions	FC Tokyo’s left winger Yasuto Tanaka has been sidelined for next Wednesday’s J1-League game. He was expected to be part of the final line-up in the must-win match against arch-rivals Kawasaki Frontale, which will take place under lights at Ajinomoto Stadium. It is believed that…	
Misinformation: Yes/No	…Tanaka’s exclusion is due to a failed drug test	…There will be a record crowd for the much-anticipated game
All conditions	Tanaka has had an up-and-down season, although his cup performances have been outstanding. He has played 150 games for Tokyo and has been in and out of the Japanese national team for a number of years. Despite Tanaka’s absence, Tokyo enters the home game as favorites. At today’s press conference the team chairman explained…	
Correction: Yes/No	…That Tanaka’s exclusion was not due to a failed drug test	…That the team still had high hopes of winning the title
All conditions	Emerging talent Ibrahim Abdallah will take Tanaka’s position in the team for the upcoming game against Kawasaki. Abdallah is at the beginning of his career which has so far proved to be formidable. He has played several games along-side Tanaka and hopes to perform well in the upcoming match. This is the last game before the cup semi-finals and the final opportunity for FC Tokyo to play a league game in front of a home crowd this season	

##### Test questionnaires

Each scenario had a corresponding test questionnaire that included ten questions: an open-ended event-summary recall question; three multiple-choice questions that assessed memory for report details; five open-ended inference questions that provided an opportunity to mention the misinformation; and one open-ended direct-inference question asking about the event’s cause (all questions are provided in Additional file 1). For methodological consistency with Autry and Duarte (2021), misinformation reliance in Experiment 1 was measured using open-ended questions. This is also consistent with much of the existing work on misinformation and the continued influence effect, which has also often used open-ended questions (Ecker et al., 2010, 2011; Johnson & Seifert, 1994; Seifert, 2002). Three of the inference questions were identical across all scenarios: “What would be a good headline for the report?”; “How could such a situation be avoided in the future?”; and “What should happen next?”. The remaining two inference questions were scenario-specific (e.g., “Why might Tanaka’s season have been described as ‘up-and-down’?”). The memory questions explicitly tested memory for details in the news reports that were unrelated to the event cause and thus the experimental manipulation (e.g., “who will FC Tokyo compete with in the upcoming game?”).

#### Procedure

Participants initially received an information sheet approved by the University of Western Australia’s Human Research Ethics Office (Ethics ID: RA/4/20/6423) and provided informed consent. Participants answered some basic demographic questions about their English proficiency, age, gender, and country of residence. Participants then read the four fictional news reports—one per experimental condition. Presentation order and assignment of reports to conditions were counterbalanced across participants using a Graeco-Latin-square design. Reading was self-paced but a minimum presentation time was enforced (set at approx. 150 ms per word). Participants were unable to revisit the reports once they had continued. After a one-minute filler-task (a word sleuth), participants completed the four questionnaires, which were presented in the same order as the reports. Lastly, participants were asked if they had put in a reasonable effort and if their data should be included in the analysis, before being fully debriefed.

### Results

#### Memory for report details

Memory was assessed only to ensure all participants included in the main analyses had encoded the reports. Memory scores were calculated across reports, based on the number of correct responses to the three multiple-choice questions per report; the maximum possible score was thus 12. Participants were required to correctly answer at least one question per news report on average (i.e., memory score ≥ 4) for their data to be included in the analyses, leading to four participants being excluded (see Participants section for more details). For the final sample (i.e., after exclusions, *N* = 276), the mean memory score was *M* = 9.12, *SD* = 2.36.

#### Scoring of misinformation reliance for open-ended responses

Reliance on misinformation was calculated by summing references made to misinformation in response to the open-ended event-summary recall question, the five open-ended inference questions, and the open-ended direct-inference question. To this end, each response was scored using values of 0, 0.5, or 1, based on a detailed written scoring guide created specifically for the data set (see Additional file 1). Any direct reference to the target misinformation or a response that implied belief in the target misinformation was scored as 1 (e.g., “Tanaka could have avoided taking drugs” or “Drugs and sports don’t mix”). Scores of 0.5 were awarded for responses that referred to the misinformation but expressed uncertainty, for example implying there was a chance that the event could be due to a reason other than the misinformation (e.g., “player was side-lined, presumably due to a failed drug test”). A score of 0 was awarded if the misinformation was mentioned but controverted (e.g., “soccer player excluded, but not due to drugs”) or if the participant did not mention the misinformation at all in their response. The maximum possible inference score was seven for each report (i.e., in each condition). A primary scorer scored all responses according to the scoring guide; ambiguous cases were additionally scored by a secondary scorer; discrepancies were resolved through discussion. To determine interrater reliability, a third scorer then scored the responses of a subsample of 36 participants, using the same scoring guide. Reliability was found to be satisfactorily high, *r* = 0.95. All scorers were blind to experimental conditions.

#### Misinformation reliance

**Fig. 1:**
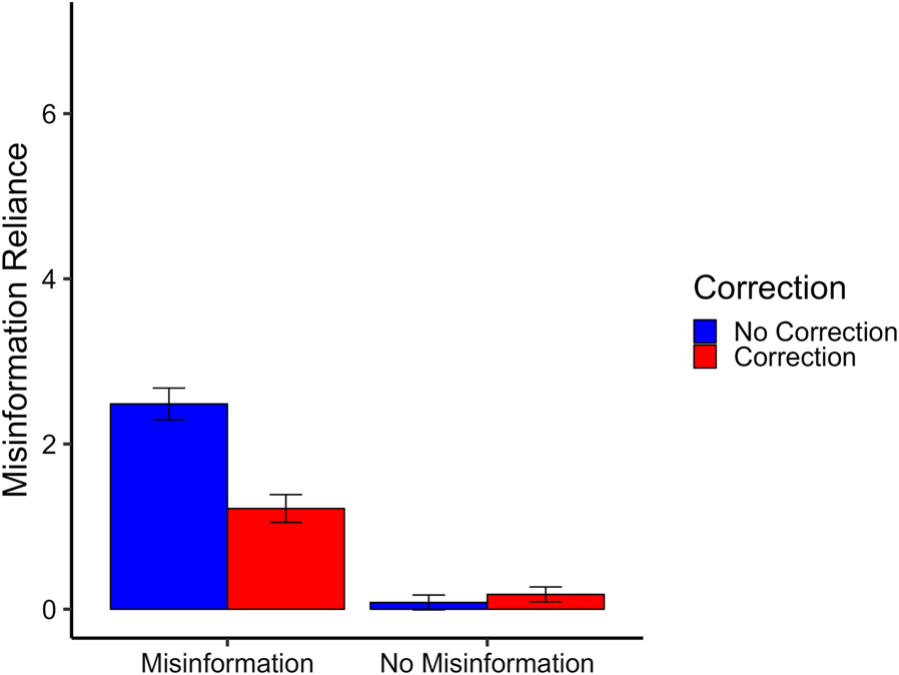
Misinformation reliance across conditions in Experiment 1. *Note.* Misinformation reliance is the average sum of misinformation reliance per condition (here: in response to open-ended inference questions; possible range 0–7). Error bars represent 95% confidence intervals

Mean misinformation reliance across conditions is shown in Fig. 1. The misinformation reliance measure included a large proportion of zeros, especially in the no-misinformation conditions. Inspection of skewness and kurtosis revealed the no-misinformation conditions violated the assumption of normal distribution with skew values ≥ 8.98 and kurtosis values ≥ 27.11. In addition, Shapiro–Wilk tests indicated violation of normal distribution for all conditions, all *W*s ≤ 0.22, all *p*s < 0.001. The deviation was considered so significant that no data transformation processes were deemed applicable. Therefore, rather than using a within-subjects ANOVA, a zero-inflated Poisson (ZIP) regression model was used for analysis. The ZIP regression model effectively addresses the high frequency of zeros often encountered in count data by concurrently modelling a discrete count distribution and the inflated number of zeros (Green, 2021; Lambert, 1992). It can therefore be considered a two-component mixture model combining a point mass at zero with a proper count distribution; zero scores may therefore come from either the point mass or the count component. The specific function used was *zeroinfl* from the R package *pscl* (Jackman, 2020; Zeileis et al., 2008); it runs a Poisson count model and a logit model for predicting excess zeros.

The two experimental factors, misinformation exposure and correction, as well as their interaction, were used to predict the number of misinformation references made by participants. The specific scenario was also included as a predictor in the model, while the repeated-measures design was accounted for by including participant ID as a predictor of both the count and the zero-inflation component. As the *zeroinfl* function expects count data and therefore cannot deal with half scores, misinformation-reference scores were multiplied by two prior to analysis. There were statistically significant main effects of misinformation exposure, β = 4.40, 95% CI [3.74, 5.07], *SE* = 0.34, *z* = 12.94, *p* < 0.001, and correction, β = 1.62, 95% CI [1.20, 2.05], *SE* = 0.22, *z* = 7.52, *p* < 0.001, indicating greater reliance on misinformation after misinformation exposure and reduced reliance after a correction. There was also a statistically significant interaction between misinformation exposure and correction, β = 1.24, 95% CI [0.85, 1.63], *SE* = 0.20, *z* = 6.23,* p* < 0.001, indicating that a correction reduced misinformation reliance only in the condition exposed to the misinformation. The specific scenario used was not a significant predictor of misinformation reliance, β = 0.002, 95% CI [− 0.04, 0.04], *SE* = 0.02, *z* = 0.10, *p* = 0.924, nor was participant ID, β < 0.001, *SE* < 0.001, *z* = 0.61, *p* = 0.542.

To establish whether a continued influence effect was present, analysis was restricted to the two conditions featuring a correction (i.e., the misinformation/correction and no-misinformation/correction conditions). The model used condition (misinformation vs. no misinformation) and scenario to predict the number of misinformation references made by participants after a misinformation-negating correction. Participant ID was again additionally included as a predictor of both count and zero-inflation components. There was a statistically significant difference between the two conditions featuring a correction, β = 1.53, 95% CI [1.22, 1.84], *SE* = 0.16, *z* = 9.54, *p* < 0.001. This demonstrates a continued influence effect: a correction following misinformation exposure did not reduce the number of misinformation references to the baseline level associated with presenting a correction in the absence of misinformation exposure. The scenario used was again not a significant predictor, β = 0.03, 95% CI [− 0.03, 0.10], *SE* = 0.03, *z* = 1.80, *p* = 0.073, nor was participant ID, β < 0.001, *SE* < 0.001, *z* =  − .29, *p* = 0.199.

The main focus of this research, however, was on the impact of a standalone correction on misinformation reliance relative to a no-misinformation/no-correction control condition. Therefore, if the results of Autry and Duarte (2021) replicate, then we would expect misinformation reliance to be higher in the no-misinformation/correction condition than the no-misinformation/no-correction control condition. To this end, a second restricted ZIP regression analysis was conducted to investigate the difference between the two no-misinformation conditions. The model used condition (correction vs. no correction) and scenario to predict the number of misinformation references made by participants after no initial exposure to the misinformation. Participant ID was again included as a predictor of both count and zero-inflation components. The model provided no evidence of a significant difference between the two conditions, β = 0.39, 95% CI [− 0.14, 0.91], *SE* = 0.27, *z* = 1.44, *p* = 0.151. Thus, there was no evidence to suggest that reading a negated correction of novel misinformation, with no initial exposure to the misinformation, increased reliance on the novel misinformation relative to a control condition. The scenario used in the news report was not a significant predictor, β = 0.11, 95% CI [− 0.15, 0.38], *SE* = 0.14, *z* = 0.82, *p* = 0.410, nor was participant ID, β = 0.002, *SE* = 0.001, *z* = − 0.89, *p* = 0.372.

**Fig. 2:**
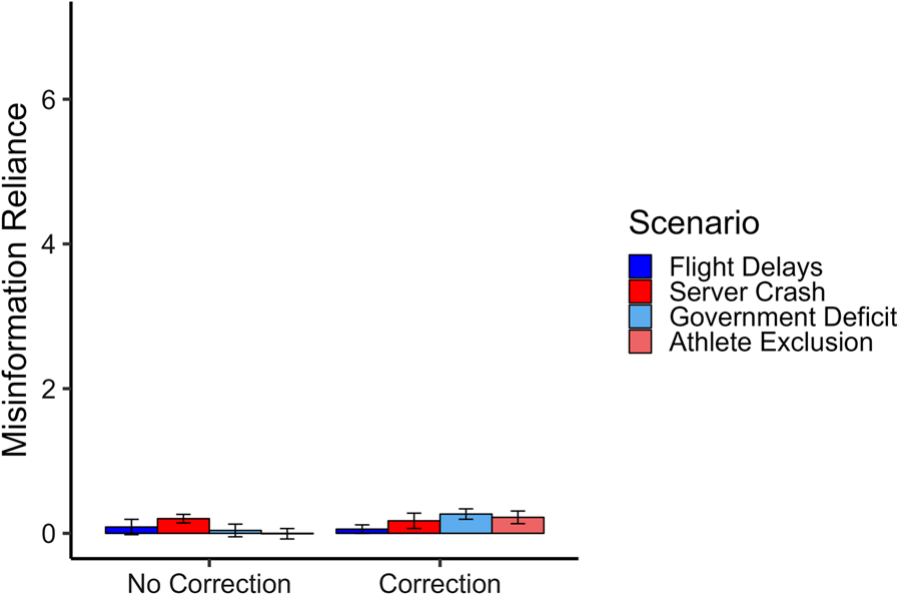
Average scenario-specific misinformation reliance in Experiment 1 following no misinformation exposure. *Note.* Misinformation reliance is the average sum of misinformation reliance per condition (here: in response to open-ended inference questions; possible range 0–7). Error bars represent 95% confidence intervals

The scenario used was not found to be a significant predictor of references made to misinformation. However, for the sake of thoroughness, an exploratory post-hoc review of the no-misinformation/correction and no-misinformation/no-correction control conditions uncovered some variation in misinformation reliance across scenarios. Mean reliance on misinformation across the two no-misinformation conditions and scenarios is shown in Fig. 2. As can be seen, only the ‘government-deficit’ scenario, and to a lesser extent the ‘athlete-exclusion’ scenario, showed a numeric increase in misinformation reliance in the no-misinformation/correction condition relative to control. Examination of these results was purely exploratory and as such no inferential statistical tests were conducted.

### Discussion

In line with our hypotheses, Experiment 1 found that corrections of novel misinformation did not lead to increased misinformation reliance, even when the corrections were presented without initial misinformation exposure. This finding is inconsistent with the results of Autry and Duarte (2021) and other previous research that suggests that corrections can backfire due to boosting claim familiarity (Pluviano et al., 2017, 2019; Skurnik et al. 2007 [unpublished]). However, the results are consistent with previous research that has not found evidence of backfire effects with either novel (Ecker et al., 2020b; Gordon et al., 2019) or potentially non-novel misinformation (Cameron et al., 2013; Ecker et al., 2017; Ecker et al., 2020c; Swire et al., 2017; Swire-Thompson et al., 2022).

In Experiment 1, we chose to use open-ended questions for methodological consistency with previous research including Autry and Duarte (2021). Although there are several benefits to this method, there are also some limitations; for instance, belief in misinformation may be underreported due to the effort of writing responses (see Connor Desai & Reimers, 2019). Furthermore, given that we are examining a familiarity-based effect, more familiarity-based procedures such as rating scales may be more sensitive than recall-based measures. We therefore switched to rating scales in Experiment 2.

## Experiment 2

Given the widespread use of corrections, even if standalone corrections of novel misinformation do not generally backfire, there could still be serious negative consequences if there are specific circumstances in which they do. Therefore, building on the results of Experiment 1, in Experiment 2 we introduced a one-week delay between reading the articles and completing the test questionnaires. Previous research has found that correction effectiveness is reduced over time (Ecker et al., 2020b; Ecker et al., 2020c; Rich & Zaragoza, 2020; Swire et al., 2017; Swire-Thompson et al., 2023), and of the few studies that have reported familiarity backfire effects, most reported these effects only after a one-week delay (Pluviano et al., 2017, 2019; Skurnik et al. 2007 [unpublished]). There are also theoretical reasons to expect that a delay may increase the risk of a correction backfiring. If corrections can inadvertently lead to increased misinformation reliance because participants rely on familiarity cues and/or fail to retrieve the correction, then introducing a delay will increase the risk of standalone corrections backfiring because familiarity is less sensitive to time delays than recollection (Yonelinas & Levy, 2002).

### Method

Experiment 2 used a 2 × 2 × 2 within-between design. Misinformation exposure (no misinformation; misinformation) and correction (no correction; correction) were within-subjects variables and test delay (no delay; delay) was a between-subjects variable. The dependent variable was reliance on misinformation, as in Experiment 1. However, Experiment 2 used 11-point rating scales rather than open-ended responses to avoid issues with zero-inflation. Memory for report details was again measured using multiple-choice questions and the experiment was administered online using Qualtrics.

#### Participants

To achieve comparable power to Experiment 1, we aimed to test 600 participants. To account for potential exclusions and drop-outs in the delay condition, 700 participants were recruited from MTurk via CloudResearch, with the one-week delay condition oversampled by 25%. Eligibility criteria were the same as for Experiment 1. There were 605 participants who completed both the study and test phases. The data were screened to exclude any participants who did not report their English proficiency as at least “good” (*n* = 1), indicated they did not reside in the U.S. (*n* = 0), or self-nominated their data to be excluded because of low effort (*n* = 0). Because there was a delay condition, participants with a memory score < 4 were not excluded. The final sample size for analysis was thus *N* = 604. The sample included 322 females, 274 males, 6 non-binary individuals, and 2 individuals preferring not to declare their gender. Participant age ranged from 22 to 89 years of age (*M* = 44.25; *SD* = 13.03). The experiment took approx. 15 min to complete; participants were paid US$3.00 for their participation, split into $1.00 for completing the study phase and $2.00 for the test phase.

#### Materials

##### News reports

The format of the reports was the same as in Experiment 1. However, we replaced the government-deficit and athlete-exclusion reports with two alternatives from the group of eight reports initially piloted (see Additional file 1). We replaced these scenarios because we believed that they induced the greatest skepticism in the correction, and we believed that high correction skepticism may contribute to a backfire effect (skepticism is explicitly examined in Experiment 3). The replacement scenarios dealt with a house fire and a car crash. These scenarios were selected based on our assessment that the corrections were less likely to induce/warrant skepticism than the removed scenarios. This initial assessment was confirmed by the results of the pilot study for Experiment 3 in which correction skepticism was explicitly measured (see Additional file 1 for full details).

##### Test questionnaires

There was again one questionnaire per scenario. Inference questions were similar to those used in Experiment 1 but were modified to use rating scales rather than being open-ended (both question types have been shown to be appropriate for measuring the continued influence effect; see Connor Desai & Reimers, 2019). Therefore, the questionnaires consisted of the same three multiple-choice questions used to assess memory in Experiment 1; five inference questions using 0–10 rating scales (e.g., “‘Black Ice Delays Flights at Houston Airport’ would be an appropriate headline for the report”—*strongly disagree*—*strongly agree*); and one direct-inference question in which participants had 100 points to assign across four potential causes, one of which was the cause suggested by the misinformation, plus a fifth option “some other cause” (e.g., “The cause of the flight delays was: (a) *black ice*; (b) *severe winds*; (c) *a bomb threat*; (d) *a staff strike*; (e) *some other cause*).

##### Procedure

The procedure was almost identical to Experiment 1. Experiment 2 differed in that participants in the delay condition did not complete the filler task; the study phase ended after they read the four reports. Participants were invited back one week later to complete the test phase.

### Results

#### Memory for report details

As in Experiment 1, memory scores were calculated based on the number of correct responses to the multiple-choice questions, with a maximum possible score of 12. For the final sample (i.e., after exclusions, *N* = 604), the overall mean memory score was *M* = 7.60, *SD* = 3.38. As expected, memory scores were significantly lower in the delay condition (*M* = 5.52, *SD* = 3.00) than the no-delay condition (*M* = 9.61, *SD* = 2.36), *t*(560.03) = 18.60, *p* < 0.001, *d* = 1.52, 95% CI [1.30, 1.75].

#### Scoring of misinformation reliance for rating scales

Reliance on misinformation was calculated for each condition by averaging across responses to the five rating scales, plus the direct-inference question. Because the ratings were on a 0–10 scale, and participants assigned 100 points on the direct-inference question, responses for the direct-inference question were divided by 10 prior to averaging. This meant that all six questions and the total score were on a 0–10 scale and were then averaged to make a composite score.

#### Misinformation reliance

**Fig. 3:**
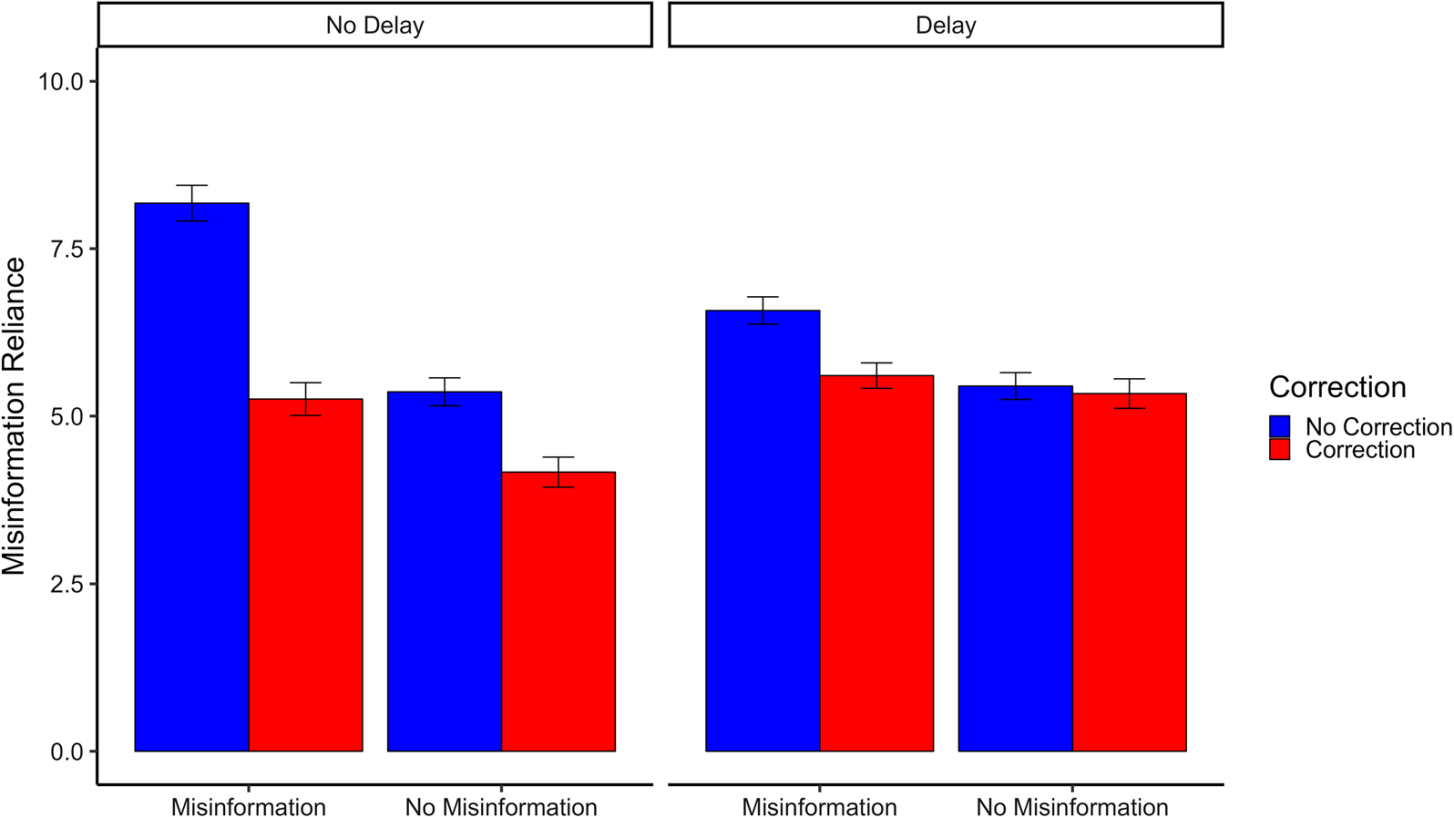
Average misinformation reliance across conditions in Experiment 2. *Note.* Misinformation reliance is average level of misinformation reliance across the rating scale and direct inference questions (possible range 0–10). Error bars represent 95% confidence intervals

Mean misinformation reliance across conditions is shown in Fig. 3. A 2 × 2 × 2 mixed ANOVA was used to examine the effect of misinformation exposure (no misinformation; misinformation), correction (no correction; correction), and delay (no delay; delay) on misinformation reliance. Results for the overall mixed ANOVA, as well as follow-up 2 × 2 within-subjects ANOVAs separated by delay condition are presented in Table 2.

**Table 2:** Experiment 2 ANOVA results: overall and split by delay condition

Predictor	*df*	*F*	*p*	η_p_^2^	95% CI
*Overall*					
Delay	1, 602	140.15	< 0.001	0.19	[0.14, 0.24]
Misinformation	1, 602	271.33	< 0.001	0.31	[0.25, 0.37]
Correction	1, 602	252.02	< 0.001	0.30	[0.24, 0.35]
Delay × misinformation	1, 602	60.68	< 0.001	0.09	[0.05, 0.14]
Delay × correction	1, 602	85.76	< 0.001	0.12	[0.08, 0.17]
Misinformation × correction	1, 602	71.12	< 0.001	0.11	[0.06, 0.15]
Delay × misinformation × correction	1, 602	8.10	0.005	0.01	[0.00, 0.04]
*No delay*					
Misinformation	1, 307	236.28	< 0.001	0.43	[0.36, 0.50]
Correction	1, 307	295.96	< 0.001	0.49	[0.42, 0.56]
Misinformation × correction	1, 307	56.93	< 0.001	0.16	[0.09, 0.23]
*Delay*					
Misinformation	1, 295	51.46	< 0.001	0.15	[0.08, 0.22]
Correction	1, 295	23.64	< 0.001	0.07	[0.03, 0.14]
Misinformation × correction	1, 295	17.92	< 0.001	0.06	[0.02, 0.12]

In the overall 2 × 2 × 2 mixed ANOVA, there were significant main effects of misinformation exposure, with greater misinformation reliance if exposed to misinformation; a main effect of correction, with lower misinformation reliance following a correction; and a main effect of delay, with greater misinformation reliance after a one-week delay. However, these were qualified by significant two-way interactions between misinformation exposure and correction, correction and delay, and misinformation exposure and delay, as well as a significant three-way interaction between misinformation exposure, correction, and delay. This showed that corrections reduced reliance on misinformation, particularly after no-misinformation exposure, although the correction’s efficacy did not last over time.

To further investigate these interactions, the data were split based on delay, and two separate within-subjects ANOVAs were conducted with misinformation exposure and correction as predictors. In the no-delay condition, there were significant main effects of misinformation exposure and correction, as well as a significant interaction. In the delay condition, there were also significant main effects of misinformation exposure and correction, as well as a significant interaction.

Further follow-ups using paired *t*-tests were then used to test for any potential correction backfire effects. When there was no delay, corrections significantly reduced misinformation reliance both when participants were exposed to misinformation, *t*(307) = 15.70, *p* < 0.001, *d* = 0.89, 95% CI [0.78, 1.02], and when they were not, *t*(307) = 8.37, *p* < 0.001, *d* = 0.48, 95% CI [0.37, 0.60]. However, the misinformation exposure by correction interaction occurred because the effect of corrections was larger following misinformation exposure. After a delay, corrections led to significantly lower misinformation reliance following misinformation exposure, *t*(295) = 6.77, *p* < 0.001, *d* = 0.39, 95% CI [0.30, 0.50], but there was no significant impact of corrections when misinformation had not previously been presented, *t*(295) = 0.71, *p* = 0.477, *d* = 0.04, 95% CI [− 0.07, 0.15], with Bayes factors showing there was strong evidence in favor of the null, *BF*_01_ = 11.94 (i.e., the data were 11.94 times more likely to have occurred under the null hypothesis). Cumulatively, these results showed that corrections were generally effective at reducing misinformation reliance but, after a one-week delay, they did not significantly reduce misinformation reliance if participants had not been initially exposed to misinformation. Crucially, there was again no evidence of standalone corrections backfiring.

For the sake of completeness, to test for a continued influence effect, the data were again split based on delay, and paired *t*-tests were then used to compare the misinformation/correction condition and the no-misinformation/correction conditions. In the no-delay condition, there was a significant difference, with lower misinformation reliance when no misinformation was initially presented, *t*(307) = 6.44, *p* < 0.001, *d* = 0.37, 95% CI [0.25, 0.49], indicating the presence of a continued influence effect relative to a standalone correction.^4^ However, in the delay condition, the difference was not statistically significant, *t*(295) = 1.89, *p* = 0.060, *d* = 0.11, 95% CI [0.00, 0.22], indicating there was no continued influence effect after a one-week delay.

### Discussion

As in Experiment 1, the results for Experiment 2 did not show any evidence of corrections backfiring. The effectiveness of corrections did wane following a delay (Paynter et al., 2019; Rich & Zaragoza, 2020), to the extent that they did not significantly reduce misinformation reliance if participants had not previously been exposed to misinformation. However, consistent with previous research (Ecker et al., 2020a; Rich & Zaragoza, 2020; Swire et al., 2017), even after a delay there was no indication that the corrections backfired and led to increased misinformation reliance. We also found that misinformation reliance was substantially higher following a delay, even in the no-misinformation conditions. Even though pilot testing showed that the causal misinformation used was not stereotypical (see Additional file 1; Table S1), the causes were still considered plausible by participants (stereotypical rating range *M* = 4.20–5.39 on a 0–10 scale). It is possible that after a one-week delay, participants’ reduced memory could have led to them relying more upon the plausibility of the misinformation rather than their memory of the event reports in their judgements. The plausibility of the causal misinformation may also explain why we did not find a continued influence effect, which is rare but not unprecedented (e.g.,Ecker & Antonio, 2021; Ecker & Rodricks, 2020). That is, if participants thought that the causal misinformation was plausible, then after a delay they may have been more willing to endorse it when responding to the rating scales, even if they had not previously been exposed to it. This higher baseline in the no-misinformation conditions may explain why we did not see a continued influence effect in the delay condition.

## Experiment 3

Another factor that may cause corrections to backfire is skepticism in the correction. Previous research has highlighted the key role of credibility for correction effectiveness (Buczel et al., 2022; Connor Desai et al., 2020; Ecker & Antonio, 2021; Guillory & Geraci, 2010, 2013; O’Rear & Radvansky, 2020). If participants are skeptical of the correction or its source, then presenting a standalone correction without initial misinformation exposure may backfire and increase misinformation reliance, as illustrated by the response to former U.S. President Nixon’s infamous “I am not a crook” utterance (Holtgraves & Grayer, 1994) or Queen Gertrude’s statement in Hamlet that “The lady doth protest too much, methinks” (Shakespeare, 1994, 3.2.330). To explore this, Experiment 3 included a manipulation of skepticism—implemented by selecting scenarios that induced low versus high levels of skepticism—to assess the effects of a standalone correction when there was (versus was not) reason to be skeptical about the correction and its source. For example, a correction from the police regarding the cause of a car crash may not induce any skepticism, whereas people may be more skeptical if a correction comes from a government spokesperson regarding the cause of a government budget deficit. It was expected that a standalone correction would have less impact—and would potentially backfire—in case of high skepticism relative to low skepticism.

### Method

Experiment 3 used a 2 × 2 within-subjects design, with correction (no correction; correction) and skepticism (low skepticism; high skepticism) as independent variables. Unlike Experiments 1 and 2, in Experiment 3 there were no conditions with initial misinformation exposure. The dependent variable was reliance on misinformation, which was measured using a combination of open-ended and rating-scale questions. Memory for report details was again measured using multiple-choice questions and the experiment was administered online using Qualtrics.

#### Participants

The design of Experiment 3 matched that of Experiment 1, and therefore we aimed to recruit the same number of participants (i.e., at least 200 participants); 280 participants were recruited to account for potential exclusions. The data were screened to exclude any participants who did not report their English proficiency as at least “good” (*n* = 0), indicated they did not reside in the U.S. (*n* = 0), self-nominated their data to be excluded because of low effort (*n* = 0), or did not meet the minimum memory score to ensure adequate encoding of materials (*n* = 4). The final sample size for analysis was thus *N* = 276. The sample included 132 females, 142 males, 1 non-binary individual, and 1 individual who preferred not to declare their gender. Participant age ranged from 22 to 76 years of age (*M* = 39.55; *SD* = 11.17). The experiment took approx. 15 min to complete; participants were paid US$2.50 for their participation.

#### Materials

##### News reports

The same format of reports was again used, although none of the reports presented initial misinformation. To select four scenarios for inclusion, an independent sample of *N* = 50 participants provided skepticism ratings for the corrections presented in the six scenarios used across Experiments 1 and 2 (see Additional file 1; Table S1). We then selected the two reports with the highest levels of skepticism in the correction (*M* = 6.22 on 0–10 scale), namely the government-deficit and athlete-exclusion scenarios used in Experiment 1, and the two reports with the lowest levels of skepticism in the correction (*M* = 3.2 on 0–10 scale), the house-fire and car-crash scenarios used in Experiment 2, for the high and low skepticism conditions, respectively. The skepticism difference was significant, *t*(49) = 8.73, *p* < 0.001, *d* = 1.23, 95% CI [0.86, 1.60].

##### Test questionnaires

There was again one questionnaire per scenario. The memory questions were the same as in Experiments 1 and 2. The five rating-scale inference questions from Experiment 2 were again used. However, for comparability with Experiment 1, the two open-ended questions from Experiment 1, namely the event-summary recall question and the direct-inference question, were also used (i.e., 7 total inference questions).

#### Procedure

The procedure was identical to Experiment 1, with one exception. Because each report was assigned to either the low or high skepticism condition based on pilot-testing, skepticism was not randomized. Each participant read two high skepticism and two low skepticism reports. Display order and correction condition (no correction; correction) were randomized, such that each participant read one event report per condition (four event reports total).

### Results

#### Memory for report details

Memory scores for the report details were comparable to Experiments 1 and 2. For the final sample (i.e., after exclusions, *N* = 276), the mean memory score was *M* = 8.92, *SD* = 1.94.

#### Scoring of misinformation reliance

Reliance on misinformation for the open-ended event-summary recall and direct-inference questions were independently coded by two coders (*r* = 0.93), with disagreements resolved via discussion. Coders were blind to correction conditions; however, because the level of skepticism was scenario-specific, this could be inferred. Open-ended responses were scored using values of 0, 0.5, and 1, based on the same scoring guide developed for Experiment 1. For the rating scales, overall scores were created by averaging across the five items.

#### Misinformation reliance

Because two different forms of response type were used in Experiment 3, rating scales and open-ended responses, we report these response types separately to examine whether the results were consistent or differed.^5^

##### Open-ended responses

As in Experiment 1, the high frequency of zeros meant the data for the open-ended responses were not normally distributed (Shapiro–Wilk test for all conditions, all *W*s ≤ 0.61, all *p*s < 0.001). Therefore, we again used ZIP regression, with the repeated-measures design accounted for by including participant ID as a predictor. Unlike the analyses for Experiment 1, scenario was not included as a predictor because including it led to issues with model fit (singular fit). The results showed a significant main effect of correction, β = 0.49, 95% CI [0.12, 0.86], *SE* = 0.19, *z* = 2.62, *p* = 0.009, which was qualified by the predicted correction by skepticism interaction, β = 0.56, 95% CI [0.06, 1.07], *SE* = 0.26, *z* = 2.18, *p* = 0.029. The main effects of skepticism, β = 0.28, 95% CI [− 0.07, 1.63], *SE* = 0.18, *z* = 1.55, *p* = 0.121, and participant ID, β = 0.001, *SE* = 0.001, *z* = 1.23, *p* = 0.218, were not significant. To further investigate the correction by skepticism interaction, follow-up ZIP regressions were conducted separately for the low and high skepticism conditions, with correction and participant ID entered as predictors. In the low-skepticism condition, there were no significant effects of correction, β = 0.14, 95% CI [− 0.20, 0.49], *SE* = 0.18, *z* = 0.80, *p* = 0.423, or participant ID, β = 0.002, *SE* = 0.002, *z* = 1.23, *p* = 0.219. However, in the high-skepticism condition, there was a significant effect of correction, β = 0.44, 95% CI [0.07, 0.81], *SE* = 0.19, *z* = 2.33, *p* = 0.020, with corrections leading to increased misinformation reliance, demonstrating a backfire effect. There was again no significant effect of participant ID, β ≤ 0.001, *SE* = 0.001, *z* = 0.59, *p* = 0.558.

##### Rating scales

For the rating scales, we used a within-subjects ANOVA with correction and skepticism as predictors. There was a significant main effect of correction, *F*(1, 275) = 33.32, *p* < 0.001, η_p_^2^ = 0.11, 95% CI [0.05, 0.18], which was again qualified by the predicted correction by skepticism interaction, *F*(1, 275) = 14.95, *p* < 0.001, η_p_^2^ = 0.05, 95% CI [0.01, 0.11], indicating that corrections were more effective for the low-skepticism condition than the high-skepticism condition. The main effect of skepticism was not significant, *F*(1, 275) = 1.22, *p* = 0.271, η_p_^2^ = 0.004, 95% CI [0.00, 0.03]. Follow-up paired *t-*tests confirmed that corrections significantly reduced misinformation reliance in the low-skepticism condition, *t*(275) = 7.84, *p* < 0.001, *d* = 0.47, 95% CI [0.35, 0.61], but had no significant effect in the high-skepticism condition, *t*(275) = 1.02, *p* = 0.308, *d* = 0.06, 95% CI [− 0.06, 0.18]. There was again no evidence of a standalone correction backfiring in the high-skepticism condition, with Bayes factors showing there was moderate evidence in favor of the null, *BF*_01_ = 8.86 (i.e., the data were 8.86 times more likely to have occurred under the null hypothesis).

### Discussion

**Fig. 4:**
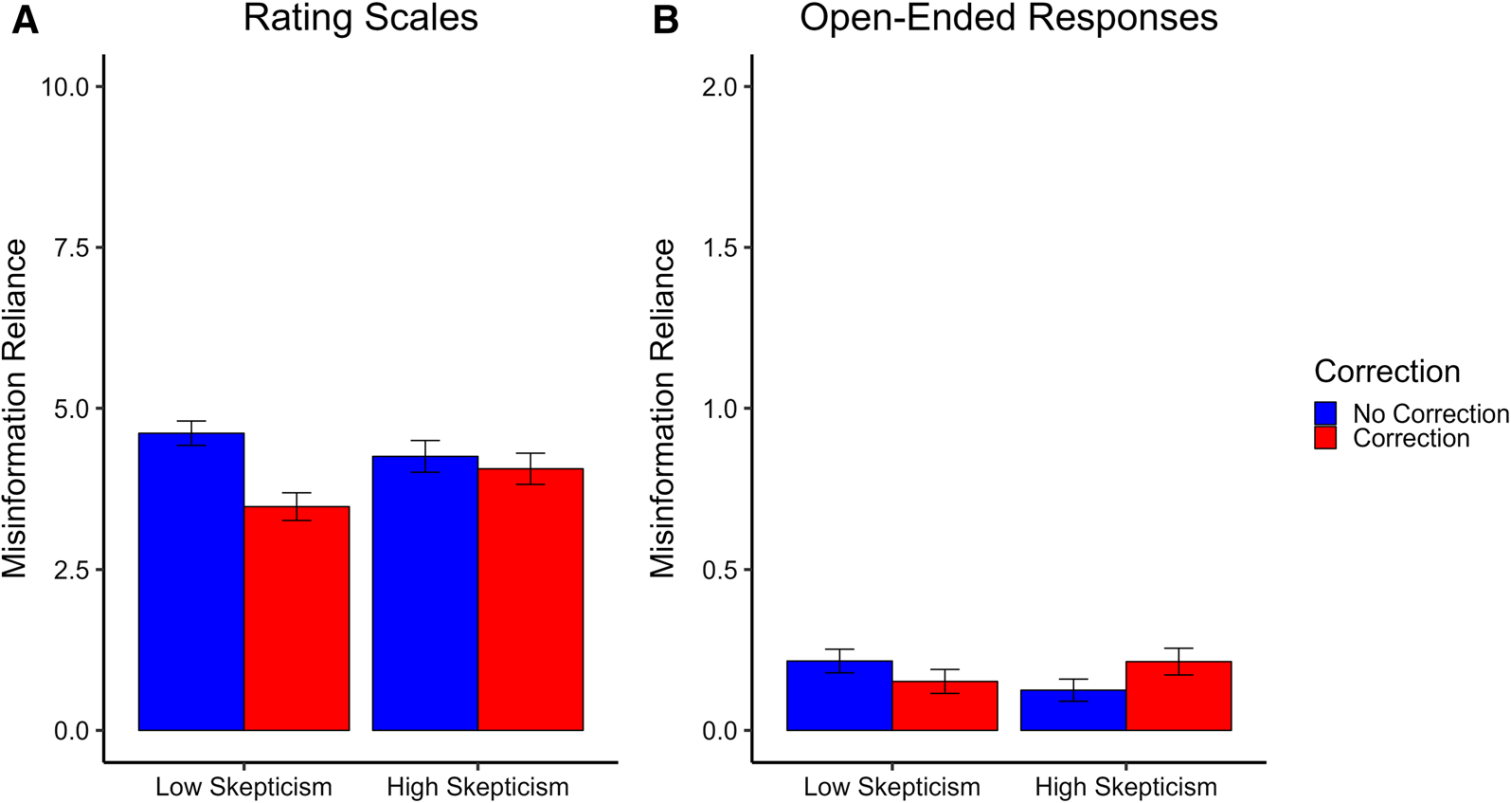
Misinformation Reliance in Experiment 3. *Note.* For the rating-scale questions misinformation reliance was averaged across items (possible range 0–10). For the open-ended responses, misinformation reliance is the average sum of misinformation reliance per condition (possible range 0–2). Error bars represent 95% confidence intervals

The results of Experiment 3 showed that skepticism in the correction can indeed impact its effectiveness, in line with previous research investigating the trustworthiness of correction sources (Buczel et al., 2022; Connor Desai et al., 2020; Ecker & Antonio, 2021; Guillory & Geraci, 2010, 2013; O’Rear & Radvansky, 2020). In the analysis of open-ended responses, presenting a standalone correction in the high-skepticism condition led to a significant increase in misinformation reliance. This was also evident from reading some of the participants’ open-ended responses (e.g., “they say it [the government deficit] was not due to the sports arena, so it is probably due to the sports arena.”). This suggests that when participants are skeptical of a correction source, a standalone correction may backfire and increase belief in misinformation. However, in the analysis of rating scales, there was no evidence of a backfire effect, even in the high-skepticism condition; this suggests that backfire risk may also depend on the method used to measure misinformation reliance (Fig. 4).

## General discussion

The results of the present study demonstrate that corrections of causal event misinformation are generally unlikely to backfire due to familiarity, even when they are presented to participants who have not previously been exposed to the negated misinformation (i.e., when the misinformation is entirely novel). In Experiment 1, we did not find evidence of a standalone correction backfiring, despite the causal misinformation being rated low on stereotypicality, suggesting that the corrections were truly unlicensed—a factor emphasized by Autry and Duarte (2021) as conducive for familiarity backfire effects to emerge. Moreover, we failed to observe any backfire even after a one-week delay (Experiment 2), which theoretically makes familiarity backfire effects more likely to emerge. When we selected scenarios that induced skepticism regarding the correction (Experiment 3), findings were mixed. With rating scales, there was no evidence of standalone corrections backfiring, but there was evidence of a backfire effect when open-ended responses were analyzed separately. This suggests that if people are skeptical of a correction, they may increase their belief post-correction. For example, if someone is generally skeptical of local government, then a correction from a city spokesperson may lead them to increase their belief in information explicitly negated by the spokesperson, particularly if the correction is seen to be self-serving. However, it should be emphasized that this backfire effect was clearly driven by skepticism, not familiarity, and as such, the observed effect is reminiscent of worldview backfire effects.

The findings of the present study should help alleviate many of the concerns raised about corrections inadvertently increasing misinformation belief via increased familiarity (Autry & Duarte, 2021; Schwarz & Jalbert, 2020). Indeed, the results from Experiments 2 and 3 suggest that standalone corrections may even be beneficial, particularly immediately after a correction and when correction skepticism is likely to be low. In other words, standalone corrections may lead to reduced levels of misinformation reliance relative to control conditions in which neither misinformation nor a correction are presented, in line with previous research (Ecker et al., 2020c; Gordon et al., 2019). This is consistent with previous work by Ecker et al. (2017) that examined the impact of repeating misinformation within a correction and found that repetition did not have any deleterious effects and instead increased the effectiveness of the correction (also see Kemp et al., 2022a, 2022b; Wahlheim et al., 2020). Ecker et al. attributed this beneficial effect to enhanced salience of the correction: If the correction explicitly mentions the to-be-corrected misinformation, this will make it clearer what the correction target is, and will allow for co-activation of misinformation and correction representations, which has been postulated as conducive to memory updating and knowledge revision (Kendeou et al., 2014). The findings of Brashier et al. (2020) may also provide some insight into why standalone corrections may not lead to a familiarity backfire effect. Brashier et al. found that prompting participants to consider the accuracy of statements prevented repeated exposure to the statements leading to an illusory truth effect. Therefore, it is plausible that if novel misinformation is encountered within the context of a correction, this may also prompt participants to consider the accuracy of the statement, which may in turn reduce the risk that misinformation exposure will lead to familiarity-driven effects (although the benefits of an accuracy prompt may decrease overtime, see Nadarevic & Erdfelder, 2014).

It is also possible that some forms of standalone corrections would be considerably more effective than the results of the current study indicate: In all three experiments, the corrections used were simple negations, which do not provide an alternative cause or detailed explanation—factors that have consistently been found to enhance the effectiveness of corrections (e.g., Ecker et al., 2010; Ecker et al., 2020c; Seifert, 2002; Swire et al., 2017). However, we acknowledge that in addition to circumstances where people may be skeptical of the correction (or correction source), there may also be other times where it may be wise to avoid correcting novel misinformation. For example, it may be reasonable for communicators to not correct novel misinformation to avoid amplifying a particular misinformation source and adopting their narrative framing of an issue (see Ecker et al., 2022), even if the risk of a correction backfiring is low. In general, our findings hence suggest that even though there might be situations in which communicators may choose to not issue a correction, there is no need to be concerned about familiarity effects, because the potential familiarity boost associated with a (standalone) correction is typically not so detrimental, on average, as to overwhelm the impact of the correction.

### Limitations and future directions

We posited that the discrepancy between the results of Autry and Duarte (2021) and those of previous studies (Ecker et al., 2020a, 2020c; Gordon et al., 2019) was the oddness of the corrections used by Autry and Duarte, which resulted in a violation of Gricean maxims of communication (Grice, 1975). However, despite conducting pilot studies to ensure that materials were not perceived as overly odd, we did not directly ask participants in our experiments the extent to which they found corrections were consistent with communication maxims, nor did we experimentally manipulate oddness or consistency with communication norms within the study. Therefore, we are unable to definitively conclude whether the oddness of the standalone corrections used by Autry and Duarte and associated violation of communication norms are the reason for the discrepant findings. Additionally, Autry and Duarte only sampled undergraduates whereas our participants were recruited from MTurk without any age restrictions. Therefore, the samples included in our experiments have a broader age ranges and older average ages. Although this has the advantage of providing a more representative sample, there are known memory differences in adults over the age of 65 (Swire et al., 2017). Although adults over 65 made up a relatively small proportion of participants, their inclusion may limit direct comparisons between our findings and Autry and Duarte (2021).

Additionally, although our findings for the effect of skepticism were mixed, there is the potential that there are at least some circumstances in which skepticism will lead to a correction backfiring. In Experiment 3, the high-skepticism corrections used were a city spokesperson stating that a budget overrun was not due to stadium construction costs and an athlete’s agent saying the athlete was not excluded from a game due to a failed drug test. Although participants may have had reason to be skeptical of the corrections or question the motives of those sources (as indicated by the high skepticism ratings in the pilot test), the corrections may have still seemed reasonably believable and there was no explicit indication that the correction sources were untrustworthy. It is therefore possible that if people are sufficiently skeptical or untrusting of a correction and its source, for example in cases where a correction is not believable, seems particularly self-serving, or comes from an explicitly discredited or notoriously untrustworthy source, a more robust backfire effect may emerge, which may also generalize across measurement types. Future research would also benefit from disentangling the effects of skepticism about the content of the correction and skepticism/lack of trust regarding the correction source. This would help establish which form of skepticism is most likely to lead to a backfire effect, or whether both elements need to present for a backfire effect to emerge. Although we found that correction skepticism may lead to a backfire effect, it is worth noting that the circumstances within our experiment were designed to induce skepticism and deviated considerably from the circumstances in which fact-checks and corrections are generally used to correct misinformation. Treating corrections from self-serving or unreliable sources with skepticism may in fact lead to fully justified, rational backfire effects (also see Connor Desai et al., 2020).

Finally, given the null effect in Autry and Duarte’s Experiment 2, it is possible that the results of their first experiment were a false-positive. Given the large number of studies examining misinformation and corrections, it is inevitable that some false-positives in individual studies will occur. Spurious findings are particularly likely if studies use single-item measures and/or do not assess the reliability of their measures (Swire-Thompson et al., 2022). However, we should still expect some spurious findings to occur even if researchers are following best practices, highlighting that replicability is crucial. For example, Ecker et al. (2020b) found a familiarity backfire effect in their first experiment but then failed to replicate that effect in two follow-up experiments. The cumulative evidence across the three experiments in Ecker et al. (2020b) strongly supported the null, suggesting the results of the first experiment were likely a false-positive. With regards to the current study, we must be conscious that open-ended responses might lack *sensitivity* to backfire effects due to people not writing responses, but also might lack *specificity* in failing to reject false-positives*.* This is because unlike rating scales, the control baseline with open-ended responses is often very close to zero, and thus the only direction for scores to “move” is up. In other words, the only effect that mentioning a misinformation concept (even within a correction) can have is to increase scores.

### Conclusion

The evidence accumulated within the current study is consistent with prior evidence showing that corrections rarely backfire (for reviews, see Ecker et al., 2022; Swire-Thompson et al., 2020, 2022), and adds to the small but growing body of evidence that backfire effects do not emerge, even when corrections expose people to novel misinformation (Ecker et al., 2020b; Gordon et al., 2019). The effectiveness of standalone corrections was diminished after a one-week delay, to the point that the corrections no longer reduced misinformation reliance. However, even after a delay, standalone corrections did not increase misinformation reliance, providing no evidence of a backfire effect. There was some tentative evidence that corrections may backfire based on skepticism, although this depended on the measure used. Cumulatively, these findings are heartening, indicating that, in general, those seeking to combat misinformation can continue to use corrections as an effective tool without fear of backfire effects.

## Supplementary Information

**Additional file 1.** Supplementary materials containing pilot study results, additional analyses, and study materials.

## Data Availability

Data and materials for this study are available on the Open Science Framework: https://osf.io/9w3fg/. For all experiments, we have reported all measures, conditions, data exclusions, and how sample sizes were determined.

## Footnotes

1 As this condition did not include a correction, the misinformation appeared as regular information from the participants’ perspective—that is, they did not know it was deemed to be false. Nevertheless, the condition is referred to as the ‘misinformation/no-correction’ condition for the sake of clarity.

2 Because we predicted a null finding, we also conducted a post-hoc power analysis using G*Power 3.1, which confirmed that our final sample size (*N* = 276) provided over 90% power (1 – = 0.91) to detect an interaction effect of size = 0.20 (with = 0.05). As the effect sizes provided by most statistics programs (and thus those reported in the literature) already factor in correlation between repeated measures as they are based on sums of squares, we selected the “as in SPSS” option within G*Power, which does not require a correlation to be specified (see Bartlett, 2022). Additionally, although G*Power does not have inbuilt functionality to allow for interactions with two within-group factors, this can be accounted for by appropriately adjusting the number of measurements (see supplementary materials of Watson et al., 2021 for details).

3 Please note that the age ranges in our experiments are broader and the average ages are older than in Autry and Duarte (2021), which only included undergraduates from California State Polytechnic University.

4 Note that the continued influence effect could also be tested by comparing the misinformation/correction condition to the no-misinformation/no-correction control condition. When tested in this way, there was no evidence of a continued influence effect, *p*s ≥ .99.

5 Analyses with the two response types combined into a composite score are presented in Additional file 1.
