# Supplementary material for: Examining the replicability of backfire effects after standalone corrections
Source: Cogn Res Princ Implic. 2023 Jul 3;8:39. doi: 10.1186/s41235-023-00492-z (PMC10317933; doi:10.1186/s41235-023-00492-z)
Supplement: Supplementary file 1 — Additional file 1. Supplementary materials containing pilot study results, additional analyses, and study materials. [file 41235_2023_492_MOESM1_ESM.docx]

# Supplementary Materials

## Experiment 1 Pilot Testing

The reports were tested independently in two pilot studies to evaluate the stereotypicality of the misinformation, as well as the oddness associated with the stand-alone negation used in the no-misinformation/correction condition. Testing was conducted via Qualtrics online surveys by two separate samples of *N* = 100 MTurk workers; participants from either pilot study were ineligible to participate in the main study. In order to make the purpose and instructions of the pilot tests clear to participants, the concepts of stereotypicality and oddness were introduced through an example unrelated to the study materials. The results of the pilot studies determined the final choice of four scenarios included in Experiment 1.

The first pilot test aimed to evaluate how stereotypical the false event causes appeared. This was assessed by presenting participants with only the first section of the misinformation version of each report, and asking them to respond to three questions per scenario, using a scale of 0 to 10: “When you think about [event X], how obvious is the [cause Y]?” (*Not obvious at all – Very obvious*); “How easy or hard would it be to come up with other potential causes?” (*Very easy – Very hard*); and “[Event X] is typically caused by [cause Y].” (*Strongly agree – Strongly disagree*)*.*

The second pilot test aimed to evaluate how odd and confusing people found the negations of the event causes when they were presented as stand-alone corrections without the causes being previously mentioned. Instructions explained the concept of relevance as essential to communication norms, and that violations of communication norms can make a statement appear odd to the reader. Participants were presented with the no-misinformation/correction version of each report (omitting the final paragraph of arbitrary information after the correction). Participants were then asked to respond to two questions: “How odd was it to learn that [event X] was NOT caused by [cause Y]?” (*Not odd at all – Very odd*) and “Were you confused about why you were being told that [event X] was not caused by [cause Y]?” (*Not confused at all – Very confused*).

The pilot data were aggregated to allocate stereotypicality and oddness scores to each scenario, on a 0 (*not stereotypical/odd at all*) to 10 (*very stereotypical/odd*) scale. The reports ranged from 2.25 to 5.39 on the stereotypicality scale, and from 1.94 to 5.82 on the oddness scale. To select the reports for the main study, initially any scenario with either score ≥ 5 was excluded (*n* = 3). Then, a stereotypicality × oddness metric was calculated and the remaining scenarios were ranked on this metric. The four least stereotypical/odd scenarios were selected for inclusion in Experiment 1. All reports are provided in the event reports section below and the pilot test results can be found in Table S1.

## Experiment 3 Pilot Testing

To examine the impact of correction skepticism, the six event reports that had been used across Experiments 1 and 2 were pilot-tested using the no-misinformation/correction version. Participants rated each of the standalone corrections on three questions, all of which were on a 0-10 scale from “Strongly Disagree” to “Strongly Agree”: “I am skeptical of the correction that [cause Y] was not the actual cause of [event X]”; “I trust the [correction source] to provide accurate information in this scenario”; and “I think there is reason to be suspicious of the statement that [cause Y] was not the actual cause of [event X]”. We then selected the two event reports with the highest skepticism ratings (for the high-skepticism condition) and the two event reports with the lowest skepticism ratings (for the low-skepticism condition) to be included in Experiment 3.

## Table S1

*Pilot Test Results of Stereotypicality and Oddness (Experiment 1) and Correction Skepticism (Experiment 3)*

|  | Stereotypicality | | Oddness | | Stereotypicality  × Oddness | Correction Skepticism | |
| --- | --- | --- | --- | --- | --- | --- | --- |
| Scenario | *M* | *SD* | *M* | *SD* |  | *M* | *SD* |
| Server Crash^ab^ | 4.48 | 2.07 | 2.02 | 2.61 | 9.03 | 5.45 | 3.00 |
| House Fire^bc^ | 5.20 | 1.46 | 1.94 | 2.40 | 10.11 | 2.99 | 2.08 |
| Government Deficit^ac^ | 2.99 | 1.95 | 3.56 | 2.50 | 10.62 | 6.76 | 2.38 |
| Athlete Exclusion^ac^ | 3.96 | 2.03 | 3.83 | 2.89 | 15.18 | 5.68 | 2.50 |
| Flight Delays^ab^ | 4.20 | 1.85 | 4.03 | 3.11 | 16.90 | 4.31 | 2.20 |
| Car Crash^bc^ | 5.39 | 1.56 | 3.17 | 2.61 | 17.05 | 3.41 | 2.31 |
| Museum Closure | 3.85 | 2.00 | 4.85 | 2.54 | 18.70 |  |  |
| School Evacuation | 2.25 | 1.97 | 5.82 | 2.82 | 13.11 |  |  |

*Note.* ^a^ indicates event reports used in Experiment 1; ^b^ indicates event reports used in Experiment 2; ^c^ indicates event reports used in Experiment 3

## Experiment 3 Combined Analyses

***Scoring of Misinformation Reliance***

Reliance on misinformation for the open-ended event-summary recall and direct-inference questions were independently coded by two coders (*r* = .93), with disagreements resolved via discussion. Coders were blind to correction conditions; however, because the level of skepticism was scenario-specific, this could be inferred. Open-ended responses were scored using values of 0, 0.5, and 1, based on the same scoring guide developed for Experiment 1. Because scores for the open-ended responses were on a 0-1 scale and the rating scales were on a 0-10 scale, the open-ended responses were multiplied by 10 prior to averaging so that all seven questions and the total score were on the same 0-10 scale and could be averaged to create a composite score.

***Misinformation Reliance***

Mean combined misinformation reliance across conditions is shown in Figure S1. A within-subjects ANOVA was used to examine the effect of correction and skepticism. There was a significant main effect of correction, *F*(1, 275) = 23.63, *p* < .001, η_p_^2^ = .08, 95% CI [.03, .15], but this was qualified by the predicted correction by skepticism interaction, *F*(1, 275) = 20.35, *p* < .001, η_p_^2^ = .07, 95% CI [.02, .13]. This indicated that corrections were more effective for the low-skepticism condition than the high-skepticism condition. The main effect of skepticism was not significant, *F*(1, 275) = 0.49, *p* = .484, η_p_^2^ = .002, 95% CI [.00, .02]. Follow-up paired *t-*tests confirmed that corrections significantly reduced misinformation reliance in the low-skepticism condition, *t*(275) = 7.22, *p* < .001, *d* = 0.44, 95% CI [0.30, 0.58], but had no significant effect in the high-skepticism condition, *t*(275) = 0.07, *p* = .941, *d* = 0.00, 95% CI [-0.11, 0.13]. Most importantly, there was no evidence of a standalone correction backfiring in the high-skepticism condition, with Bayes factors showing there was strong evidence in favor of the null, *BF*_01_ = 14.79 (i.e., the data were 14.79 times more likely to have occurred under the null hypothesis).

**Figure S1**

*Misinformation Reliance for Composite Scores (Response Types Combined) in Experiment 3*


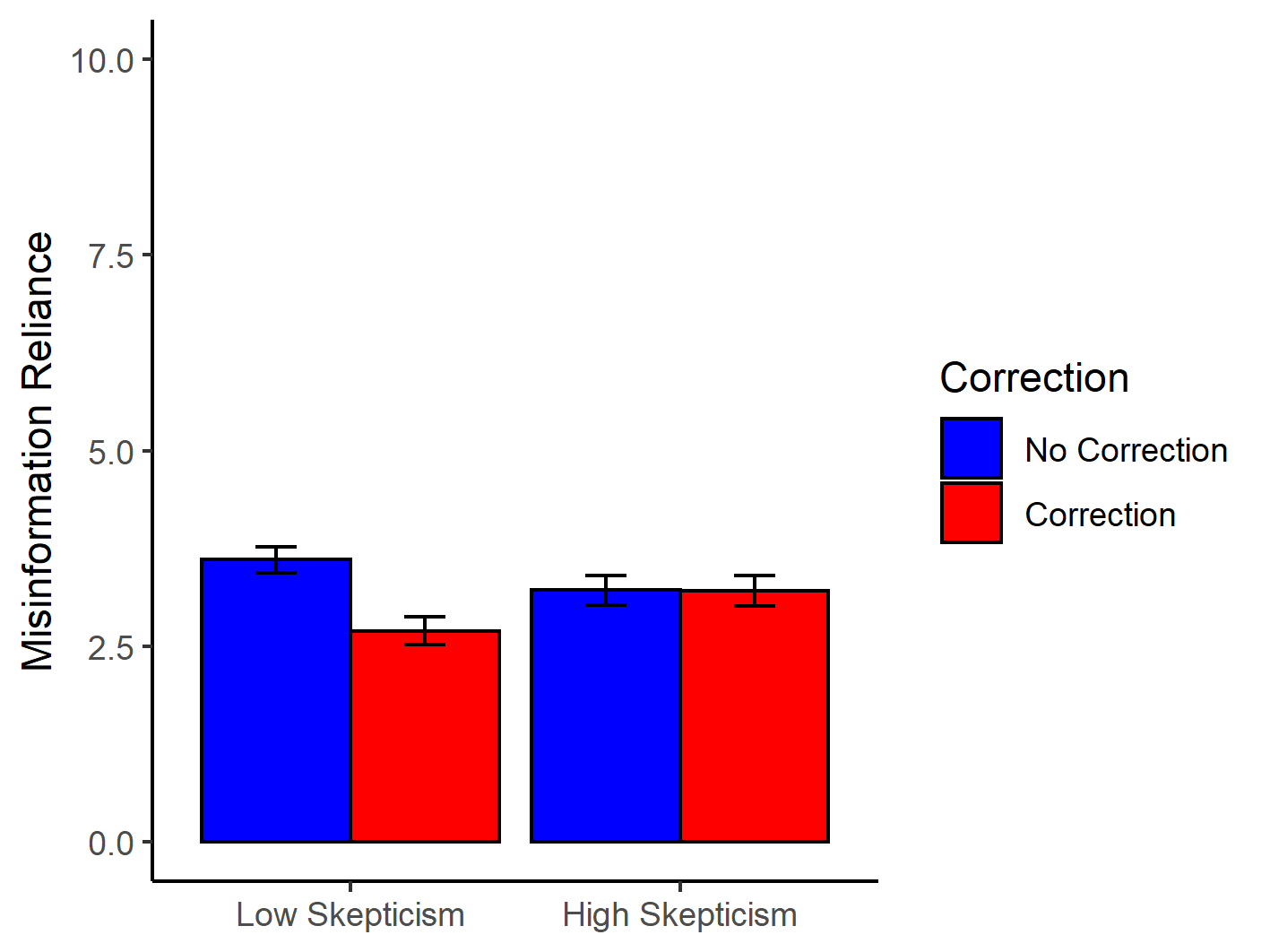


*Note.* Misinformation reliance is average level of misinformation reliance across the open ended and rating scale questions (possible range 0-10). Error bars represent 95% confidence intervals.

## Event Reports

### Delayed Flights

The holiday season has been interrupted suddenly by flight delays at George Bush Intercontinental Airport in Houston. People have been waiting for information from the airport about their delayed flights. Two women going on vacation to New York were very understanding of the delays; they spoke with our correspondent: “These things can’t be helped, we just have to wait for our flights. At least the restaurants are still open, which is great.” The two women…

*Misinformation:* …understood the delays were caused by black ice on the runways.

*No Misinformation:* …mentioned their flight had already been pushed back by 5 hours.

The airport became very busy and crowded, as the flight delays had not been communicated to all departing passengers. A group of impatient passengers demanded to speak to someone in charge to get more information. The airport’s operations manager was contacted and assured the passengers that everything was being done to resume flights; they also stated that …

*Correction:* …the delays were not caused by black ice on the runways.

*No Correction:* …the airport was handing out food vouchers to all affected passengers.

In 2019, the Houston airport was ranked as the 15^th^ busiest airport in the United States. The holiday season is the busiest time of year for most airports, so the delays come at an inopportune time. They will leave airlines frantically re-organizing to get passengers to their final destinations as soon as possible.

### Server Crash

Chaos erupted on Wall Street yesterday afternoon around 1.30pm when the computer servers of the New York Stock Exchange temporarily crashed. Stockbrokers found themselves without access to the stock exchange platform, unable to view or trade stocks. National and international clients wasted no time in contacting their brokers to get more information on the situation. It is…

*Misinformation:* …thought a cyber-attack was responsible for the incident.

*No Misinformation:* …fair to say that it was a turbulent day at the world’s largest stock market.

All trading was halted, as a team of computer technicians worked under immense pressure to rectify the situation as quickly as possible. News of the server crash spread like wildfire through social media, with #WallStShutDown trending on twitter. When trading resumed, the Dow Jones index dipped by more than 300 points. It was later clarified that…

*Correction*: …the server crash had not been caused by a cyber-attack.

*No Correction*: …all systems were stable and performing well.

Trading resumed at 2pm after a half-hour interruption, and the market quickly recovered after the initial dip, with the Dow Jones closing at just under 34,000 points. The strongest performers were technology and pharmaceutical stocks. With brokers thankful for the computer technicians and their work, they can look forward to trading normally next week.

### Athlete Sidelined

FC Tokyo’s left winger Yasuto Tanaka has been sidelined for next Wednesday’s J1-League game. He was expected to be part of the final line-up in the must-win match against arch-rivals Kawasaki Frontale, which will take place under lights at Ajinomoto Stadium. It is believed that…

*Misinformation:* …Tanaka’s exclusion is due to a failed drug test.

*No Misinformation:* …there will be a record crowd for the much-anticipated game.

Tanaka has had an up-and-down season, although his cup performances have been outstanding. He has played 150 games for Tokyo and has been in and out of the Japanese national team for a number of years. Despite Tanaka’s absence, Tokyo enters the home game as favorites. At today’s press conference the team chairman explained…

*Correction*: …that Tanaka’s exclusion was not due to a failed drug test.

*No Correction*: …that the team still had high hopes of winning the title.

Emerging talent Ibrahim Abdallah will take Tanaka’s position in the team for the upcoming game against Kawasaki. Abdallah is at the beginning of his career which has so far proved to be formidable. He has played several games along-side Tanaka and hopes to perform well in the upcoming match. This is the last game before the cup semi-finals and the final opportunity for FC Tokyo to play a league game in front of a home crowd this season.

### Government Deficit

The local government in Geelong, Australia, has run its budget into a major deficit. The quarterly budget figures are about to be published and are anticipated to reveal large shortfalls. The city will undoubtedly be required to re-assess its financial position given this emerging situation. Members of the local government have been invited to comment but have declined at this time. According to external sources, the financial deficit …

*Misinformation*: …has been caused by the city contributing significant funds to the construction of the new sports stadium, due to be completed by the end of the year.

*No Misinformation:* …will present a significant challenge for the city in the current environment.

Independent financial audit firm Hall Chadstone has been appointed to review the city’s finances. Ahead of the review being released, the auditor^[[1]](#footnote-1)^ has already disclosed that…

*Correction*: …the deficit was not caused by the city contributing any funds to the new sports stadium currently under construction.

*No Correction*: …the deficit will require some tough decisions to be made, moving forward.

Geelong was established in 1837 and proclaimed a city in 1910. The port city lies on Corio Bay, west of Melbourne, and is a significant regional center with a population of approximately 270,000. The region’s first inhabitants were the Wathaurong people, and the city’s name is derived from their name for the bay.

### Car Crash

A car accident at the intersection of Elm Hill Pike and McGavock Pike in Nashville, TN was reported to police on Saturday afternoon. The crash involved a Ford truck and a Hyundai sedan. The sedan suffered significant structural damage and was found by the police in shrubs at the side of the road. After a brief initial assessment of the incident, a crash investigator suggested the incident was…

*Misinformation:* …due to the driver of the truck running a red light.

*No Misinformation:* …a frank reminder to be safe while driving.

The 36-year-old driver of the sedan was admitted to an intensive care unit, where they are being treated for major injuries to the chest and head. The truck driver sustained significant injuries to the lower limbs and abdomen. In a later statement, an officer of the Major Crash Investigation Unit stated that…

*Correction:* …the crash was not caused by the truck driver running a red light.

*No Correction:* …the injured drivers were recovering and no longer in a critical condition.

According to Tennessee state records, the road fatality rate has increased to 5.5 deaths per 100,000 people, compared to last year’s rate of 4.9 deaths per 100,000 people. A spokesperson for the Auto Club AAA stated that they were continuing to lobby for investments in roads and driver education, and that it was everyone’s duty to keep the roads as safe as possible.

### House Fire

On Sunday night, firefighters were called to a house fire in South Seattle. The fire crew arrived promptly from Seattle Fire Station 13 and immediately assessed the situation to determine if there were people in danger inside the house. A young family of four escaped the blaze but suffered minor injuries. The fire substantially damaged the front of the home. A preliminary report of the incident suggested that the fire…

*Misinformation:* …was due to faulty wiring.

*No Misinformation:* …had started at around 9pm.

The young family of four was attended to at a local emergency room and received treatment for smoke inhalation and minor burns; they remain in hospital but are expected to be released shortly. Fire investigation officers conducted an inspection of the property; based on initial evidence they concluded that…

*Correction:* …the fire was not due to faulty wiring.

*No Correction:* …there was no immediate risk of the house collapsing.

Neighbors have been showing their support, and have donated clothes, food, and basic hygiene products. Although on this occasion the fire department was able to respond promptly, local community groups have voiced concerns about increasing emergency response times in the municipal area. A spokesperson said: “The fire crew should be commended for their quick response, as lives could have been lost in this fire. This shows how vital our emergency services are to our community.”

### Museum Closure (Pilot tested but not used)

The Historical Museum of Prague, one of Eastern Europe’s oldest and most popular museums, has been closed until further notice. The museum has an exhibition area of 25,000 square metres, and houses nearly 10,000 objects from prehistory to the 21st century. The closing left many disappointed tourists on the streets of the “Golden City”. Earlier today, a spokesperson explained that…

*Misinformation*: …the museum’s closure was caused by a structural issue with the building’s roof.

*No Misinformation*: …any purchased tickets would remain valid but could also be refunded.

The Historical Museum was due to host the popular Smithsonian Institute’s travelling exhibition “Eternal Life in Ancient Egypt”. The exhibition was much anticipated by the museum’s head curator and regular patrons. The director of the Historical Museum spoke with our correspondent and clarified that…

*Correction*: …the museum’s closure was not due to any structural issues with the building’s roof.

*No Correction*: …the special exhibition was being diverted to the Czech National Museum.

Prague boasts a vibrant arts scene, phenomenal architecture and gardens, as well as a surprisingly varied array of culinary experiences. The Golden City is therefore one of Europe’s most popular tourist destinations, attracting more than 8 million visitors annually.

### School Evacuation (Pilot tested but not used)

Approximately 500 students and staff were evacuated from the South Wing of Bronx High School of Science on Monday morning. Our correspondent interviewed the principal, Aurelia Hancock, who commented on the evacuation. The principal explained that everyone was safe and that…

*Misinformation:* …the evacuation had been triggered by a chemical spill.

*No Misinformation:* …students had very calmly followed instructions.

The 500 teachers, students, and admin staff first marshalled in the school’s emergency assembly area; everyone then gathered at Jerome Park and the adjacent local coffee shop “Delilah’s Café” until the school building was deemed safe and they were allowed back inside. Later that day it was clarified that …

*Correction:* …the evacuation had not been caused by a chemical spill.

*No Correction:* …all students had eventually returned to the building and resumed classes.

Bronx Science, as the school is known locally, was established in 1938 and moved to its current premises on West 205^th^ St in 1959. It is a selective public school that provides science-oriented education; it has been ranked as the 4^th^ best STEM school in the nation. Bronx Science graduates include eight Nobel Prize laureates and many other prominent public figures including former Executive Editor at the *New York Times*, Joseph Lelyveld.

## Experiment 1 Test Questionnaires

### Delayed Flights Questionnaire

**Summary**

Please write a brief summary of the Delayed Flights story you read earlier.

**Memory**

1. In which city were the flights delayed?
   - Atlanta
   - Los Angeles
   - Houston
   - Washington DC
2. What staff member at the airport provided information?
   - the security manager
   - the communications manager
   - the operations manager
   - the customer service manager
3. Where is Houston Airport ranked in the list of the busiest U.S. airports by passenger traffic?
   - 15^th^
   - 11^th^
   - 19^th^
   - 6^th^

**Inference**

1. Why were the two women going on vacation so understanding of the delays?
2. Why do you think the delays were described as “sudden”?
3. What would be a good headline for the report?
4. How could such a situation be avoided in the future?
5. What should happen next?

**Direct**

What do you think was the cause of the flight delays?

***Server Crash Questionnaire***

**Summary**

Please write a brief summary of the Server Crash story you read earlier.

**Memory**

1. By how many points did the Dow Jones index dip?
   - More than 50
   - More than 300
   - More than 600
   - More than 1,000
2. At what time did the server crash occur?
   - 1.30pm
   - 3pm
   - 10am
   - 11.30am
3. What stocks were among the strongest performers on the day?
   - Pharmaceutical stocks
   - Financial stocks
   - Retail stocks
   - Energy stocks

**Inference**

1. Why might investors be worried about this incident?
2. Why were the computer technicians under immense pressure?
3. What would be a good headline for the report?
4. How could such a situation be avoided in the future?
5. What should happen next?

**Direct**

What do you think was the reason for the server crash?

### Athlete Sidelined Questionnaire

**Summary**

Please write a brief summary of the Athlete Sidelined story you read earlier.

**Memory**

1. Who will FC Tokyo compete with in the upcoming game?
   - Kawasaki Frontale
   - Nagoya Grampus
   - Yokohama FC
   - Gamba Osaka
2. How many games has Tanaka played for FC Tokyo?
   - 150
   - 75
   - 200
   - 300
3. At which stadium will be match be played?
   - Ajinomoto Stadium
   - Tokyo Dome
   - Nissan Stadium
   - Fukuda Denshi Arena

**Inference**

1. Why might Tanaka’s season have been described as “up-and-down”?
2. What might fans think of Tanaka’s exclusion?
3. What would be a good headline for the report?
4. How could such a situation be avoided in the future?
5. What should happen next?

**Direct**

Why do you think Tanaka was sidelined for the upcoming game?

### Government Deficit Questionnaire

**Summary**

Please write a brief summary of the Local Government Deficit story you read earlier.

**Memory**

1. What Australian city was affected by the financial deficit?
   - Geelong
   - Warrnambool
   - Bendigo
   - Dandenong
2. What is the name of the independent financial audit firm?
   - Hall Chadstone
   - M & K
   - Johnson and Craik
   - LPJ Finance
3. What is the approximate population of the city mentioned?
   - 270,000
   - 420,000
   - 750,000
   - 110,000

**Inference**

1. Can you specify any concrete decisions that contributed to the city’s financial deficit?
2. What might residents think about the deficit?
3. What would be a good headline for the report?
4. How could such a situation be avoided in the future?
5. What should happen next?

**Direct**

What do you think is the main reason the city’s finances have run into a deficit?

## Experiment 2 Test Questionnaires

### Delayed Flights Questionnaire

**Memory**

1. In which city were the flights delayed?
   - Atlanta
   - Los Angeles
   - Houston
   - Washington DC
2. What staff member at the airport provided information?
   - the security manager
   - the communications manager
   - the operations manager
   - the customer service manager
3. Where is Houston Airport ranked in the list of the busiest U.S. airports by passenger traffic?
   - 15^th^
   - 11^th^
   - 19^th^
   - 6^th^

**Inference Scale (0 “Strongly disagree” to 10 “Strongly agree”)**

1. Passengers were annoyed because they were not informed of the black ice delaying their flights.
2. A heated runway would have avoided the flight delays.
3. “Black Ice Delays Flights at Houston Airport” would be an appropriate headline for the report.
4. Houston airport should invest in its de-icing resources to avoid such situations from re-occurring.
5. Houston airport should review its runway de-icing procedures.

**Direct Scale (100 points to allocate)**

The cause of the flight delays was:

- black ice
- severe winds
- a bomb threat
- a staff strike
- some other cause

### Server Crash Questionnaire

**Memory**

1. By how many points did the Dow Jones index dip?
   - More than 50
   - More than 300
   - More than 600
   - More than 1,000
2. At what time did the server crash occur?
   - 1.30pm
   - 3pm
   - 10am
   - 11.30am
3. What stocks were among the strongest performers on the day?
   - Pharmaceutical stocks
   - Financial stocks
   - Retail stocks
   - Energy stocks

**Inference Scale (0 “Strongly disagree” to 10 “Strongly agree”)**

1. Investors should be worried about cyber-security at the New York Stock Exchange.
2. The FBI’s cyber-crime unit should investigate the incident.
3. “Cyber-Attack Causes Crash on New York Stock Exchange” would be an appropriate headline for the report.
4. The server crash at the New York Stock Exchange could have been avoided if there was better cyber-security.
5. The New York Stock Exchange should invest in improving their cyber-security to avoid such situations re-occurring.

**Direct Scale (100 points to allocate)**

The cause of the server crash was:

- a cyber-attack
- an electrical fault
- an issue with a software update
- an unusual amount of traffic
- some other cause

### Car Crash Questionnaire

**Memory**

1. In which state did the car crash occur?
   - Tennessee
   - Oregon
   - Missouri
   - Colorado
2. What brand was the sedan?

- Hyundai
- Toyota
- Chevrolet
- Honda

1. How old was the driver of the sedan?
   - 36
   - 48
   - 24
   - 55

**Inference Scale (0 “Strongly disagree” to 10 “Strongly agree”)**

1. The victim’s family will be upset about the truck driver’s actions.
2. The truck driver should be charged over the incident.
3. “Drivers Injured After Truck Runs Red Light” would be an appropriate headline for the report.
4. The number of accidents such as this one could be reduced by more red-light cameras at intersections.
5. As a result of this incident, harsher penalties for running red-lights should be considered.

**Direct Scale (100 points to allocate)**

The car accident was caused by:

- the truck driver running a red light
- an animal running across the road
- the sedan suffering from a brake failure
- an oil spill in the intersection
- some other cause

### House Fire Questionnaire

**Memory**

1. What Fire Station responded to the house fire?

- Fire Station 13
- Fire Station 14
- Fire Station 30
- Fire Station 27

1. Who was rescued from the house fire?

- A couple and their pet dog
- A young family of four
- A bachelor
- An older woman and her cats

1. What did the neighbors do?

- They donated goods
- They called 911
- They gathered on the street
- They looted rooms at the back of the house

**Inference Scale (0 “Strongly disagree” to 10 “Strongly agree”)**

1. The family should be upset with the contractors who installed the house’s electrical wiring.
2. There should be an investigation into who was responsible for the electrical wiring.
3. “Faulty Wiring Causes Blaze” would be an appropriate headline for the report.
4. A thorough building inspection could have prevented the fire.
5. New home buyers should be encouraged to have their house’s electrical wiring checked to avoid such situations from re-occurring.

**Direct Scale (100 points to allocate)**

The fire was caused by:

- faulty wiring
- a candle
- arson
- children playing with matches
- some other cause

## Experiment 3 Test Questionnaires

### Car Crash Questionnaire

**Summary**

Please write a brief summary of the Car Crash story you read earlier.

**Memory**

1. In which state did the car crash occur?
   - Tennessee
   - Oregon
   - Missouri
   - Colorado
2. What brand was the sedan?

- Hyundai
- Toyota
- Chevrolet
- Honda

1. How old was the driver of the sedan?
   - 36
   - 48
   - 24
   - 55

**Inference Scale (0 “Strongly disagree” to 10 “Strongly agree”)**

1. The victim’s family will be upset about the truck driver’s actions.
2. The truck driver should be charged over the incident.
3. “Drivers Injured After Truck Runs Red Light” would be an appropriate headline for the report.
4. The number of accidents such as this one could be reduced by more red-light cameras at intersections.
5. As a result of this incident, harsher penalties for running red-lights should be considered.

**Direct**

What was the cause of the car accident?

### House Fire Questionnaire

**Summary**

Please write a brief summary of the House Fire story you read earlier.

**Memory**

1. What Fire Station responded to the house fire?

- Fire Station 13
- Fire Station 14
- Fire Station 30
- Fire Station 27

1. Who was rescued from the house fire?

- A couple and their pet dog
- A young family of four
- A bachelor
- An older woman and her cats

1. What did the neighbors do?

- They donated goods
- They called 911
- They gathered on the street
- They looted rooms at the back of the house

**Inference Scale (0 “Strongly disagree” to 10 “Strongly agree”)**

1. The family should be upset with the contractors who installed the house’s electrical wiring.
2. There should be an investigation into who was responsible for the electrical wiring.
3. “Faulty Wiring Causes Blaze” would be an appropriate headline for the report.
4. A thorough building inspection could have prevented the fire.
5. New home buyers should be encouraged to have their house’s electrical wiring checked to avoid such situations from re-occurring.

**Direct**

What do you think was the cause of the fire?

### Government Deficit Questionnaire

**Summary**

Please write a brief summary of the Local Government Deficit story you read earlier.

**Memory**

1. What Australian city was affected by the financial deficit?
   - Geelong
   - Warrnambool
   - Bendigo
   - Dandenong
2. What is the name of the independent financial audit firm?
   - Hall Chadstone
   - M & K
   - Johnson and Craik
   - LPJ Finance
3. What is the approximate population of the city mentioned?
   - 270,000
   - 420,000
   - 750,000
   - 110,000

**Inference Scale (0 “Strongly disagree” to 10 “Strongly agree”)**

1. Geelong residents would have reason to be upset with the officials overseeing the stadium construction.
2. There should be an investigation into the city’s construction projects.
3. “Stadium Construction Leads to Budget Blow Out” would be an appropriate headline for the report.
4. Better management of construction projects by the local government would have prevented the budget deficit.
5. An independent auditor to monitor local-government construction projects could help avoid such situations from re-occurring.

**Direct**

What do you think is the main reason the city’s finances have run into a deficit?

### Athlete Sidelined Questionnaire

**Summary**

Please write a brief summary of the Athlete Sidelined story you read earlier.

**Memory**

1. Who will FC Tokyo compete with in the upcoming game?
   - Kawasaki Frontale
   - Nagoya Grampus
   - Yokohama FC
   - Gamba Osaka
2. How many games has Tanaka played for FC Tokyo?
   - 150
   - 75
   - 200
   - 300
3. At which stadium will be match be played?
   - Ajinomoto Stadium
   - Tokyo Dome
   - Nissan Stadium
   - Fukuda Denshi Arena

**Inference Scale (0 “Strongly disagree” to 10 “Strongly agree”)**

1. Fans will be disappointed in Yasuto Tanaka’s actions.
2. Tanaka should be given a lengthy suspension.
3. “Tanaka Out After Failed Drug Test” would be an appropriate headline for the report.
4. Closer monitoring of players would have reduced the chances of Tanaka missing the match.
5. Education for players about the dangers and downsides of performance enhancing drugs could help prevent similar incidents in the future.

**Direct**

Why do you think Tanaka was sidelined for the upcoming game?

1. Please note that in Experiment 3, for both the pilot testing and main study, “the auditor has already disclosed that…” was changed to “a city spokesperson said that…” [↑](#footnote-ref-1)
